# Pineal and leptomeningeal metastases from a parotid carcinoma ex pleomorphic adenoma

**DOI:** 10.1259/bjrcr.20150279

**Published:** 2016-01-19

**Authors:** Olivia Francies, Dimitrios Paraskevopoulos, Ganesalingam Narenthiran, Kim Piper, Amen Sibtain, Curtis Offiah, Anant S Krishnan

**Affiliations:** ^1^ Imaging Department, Barts Health NHS Trust, London, UK; ^2^ Department of Neurosurgery, Barts Health NHS Trust, London, UK; ^3^ Department of Cellular Pathology, Barts Health NHS Trust, London, UK; ^4^ Department of Radiation Oncology, Barts Health NHS Trust, London, UK

## Abstract

Carcinoma ex pleomorphic adenoma (CEPA) is an uncommon complication of an untreated pleomorphic adenoma (PA), but one that has a life-threatening significance. This case report documents the clinical, radiological and histopathological features of an extremely rare case of biopsy-proven pineal metastasis, with cerebellopontine and leptomeningeal spread, from CEPA of the parotid gland in spite of the patient having undergone parotidectomy, ipsilateral neck dissection and adjuvant radiotherapy. In spite of the current surgical and oncological treatment of CEPA, the rates of recurrence and distant metastases are high, with a subsequently poor prognostic outcome in most patients. Distant spread is usually to the bones and the lungs; however, more unusual locations have been documented. Our finding of pineal metastasis from CEPA has not previously been reported in the literature. Although this is a rare complication of an unusual condition, the aggressive behaviour of these malignancies warrants close clinical follow-up, with a low threshold for re-imaging and investigation if indicated.

## Clinical findings

**Figure 1.**
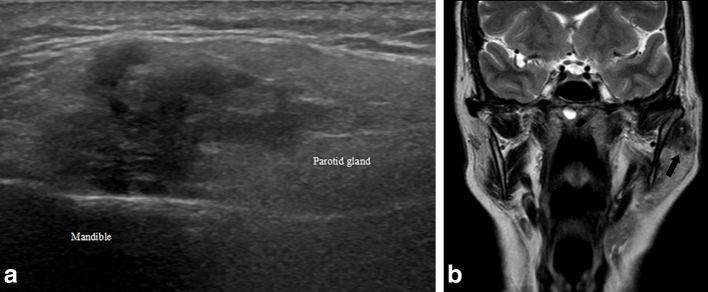
(a) Ultrasound image (longitudinal) demonstrating a poorly defined, irregular, hypoechoic lesion that corresponds to the primary parotid tumour, within the superficial parotid gland extending towards the deeper lobe. (b) Coronal *T*_2_ weighted craniofacial MRI image demonstrating a low signal lesion within the left parotid gland, adjacent to the mandible (black arrow), which corresponds to the primary parotid tumour.

**Figure 2.**
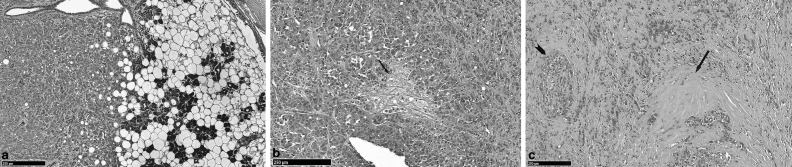
(a) H and E-labelled section (10× magnification) of the parotid tumour demonstrating tumour invasion of the normal serous acini of the parotid gland. The tumour cells have eccentrically-placed nuclei consistent with plasmacytoid morphology seen frequently in PAs. (b) H and E-labelled section (10× magnification) of the parotid tumour demonstrates an area of myxoid stroma (black arrow), which is further evidence that the tumour arose from a pre-existing PA. (c) H and E-labelled section (10× magnification) of the parotid tumour showing hyalinization (black arrow), which occurs more frequently in PAs that undergo malignant transformation. Extensive perineural invasion (black arrowhead) is also shown, highlighting the aggressiveness of the tumour. (All histological images were provided courtesy of Dr Reshma Agrawal, Specialist Registrar in Oral Pathology, Division of Cellular Pathology at The Royal London Hospital, London, UK.) H and E, haematoxylin and eosin; PA, pleomorphic adenoma.

We present the case of a 62-year-old female with a high-grade parotid carcinoma ex pleomorphic adenoma (CEPA) that was initially treated with left parotidectomy and ipsilateral neck dissection. The patient originally presented with a 1-year history of a left parotid region mass and co-existing ipsilateral facial nerve palsy, with the diagnosis confirmed using ultrasound, MRI (Figure 1a,b) and core biopsy of the lesion (Figure 2a,c). At the time of surgery, there was no evidence of direct extracapsular spread of the tumour. However, multiple anterior cervical lymph nodes were infiltrated and the tumour was staged at pT4apN2bM0. Adjuvant radiotherapy was commenced immediately after surgery.

The patient was well at initial follow-up, with no clinical or radiological evidence of recurrence. However, approximately 6 months after completing radiotherapy, she started experiencing tinnitus as well as feelings of “drunkenness”. Fine needle aspiration cytology was performed on a suspicious left supraclavicular fossa lymph node during an outpatient visit to the head and neck one-stop clinic. This demonstrated features consistent with a poorly differentiated metastatic malignancy, likely adenocarcinoma. An audiogram performed at the same time confirmed complete hearing loss in the left ear.

**Figure 3.**
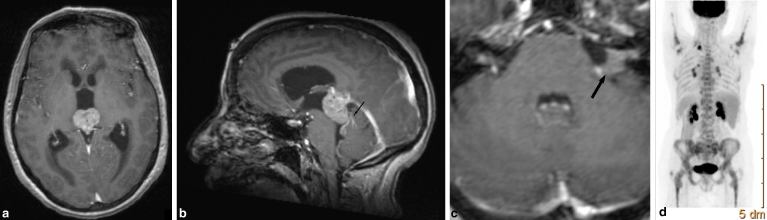
(a) Axial gadolinium-enhanced *T*_1_ weighted MRI demonstrating the large pineal region tumour obstructing the cerebral aqueduct and giving rise to acute hydrocephalus. (b) Sagittal gadolinium-enhanced *T*_1_ weighted MRI demonstrating the pineal region tumour as well as contrast enhancement along the folia of the superior cerebellar parenchyma (black arrow) in keeping with leptomeningeal spread of the tumour. (c) Axial gadolinium-enhanced *T*_1_ weighted MRI of the posterior fossa demonstrating the left cerebellopontine angle deposit (black arrow) that extends into the left internal auditory meatus. (d) Whole body ^18^F-FDG positron emission tomography scan (posterior view) demonstrating the FDG-avid left supraclavicular fossa lymph nodes, as well as metabolically active bilateral hilar lymph nodes and widespread metastatic bony infiltration. FDG, 2-deoxy-2-[^18^F]-fluoro-D-glucose.

A subsequent urgent MRI demonstrated an avidly enhancing, large pineal region tumour with resulting obstructive hydrocephalus (Figure 3a). Additionally, abnormal leptomeningeal enhancement was shown extending from the mass over the folia of the superior cerebellar parenchyma (Figure 3b). A small, discrete, enhancing lesion was also present in the left cerebellopontine angle (Figure 3c). The MRI also showed disease progression within the left supraclavicular fossa, although there was no evidence of recurrence within the parotid bed. This was confirmed on subsequent whole body 2-deoxy-2-[^18^F]-fluoro-D-glucose (^18^F-FDG) positron emission tomography/CT (PET/CT) imaging, which also revealed multiple bone metastases and bilateral metabolically active hilar lymph nodes, also suspicious for metastases (Figure 3d).

The patient was discussed at the head and neck oncology and neuro-oncology multidisciplinary meetings. The most likely differential diagnoses for the pineal mass at this stage included metastases, a high-grade pineal parenchymal tumour or a germ cell tumour. Metastases from a primary tumour other than a parotid tumour was thought to be more likely, as the pattern of intracranial spread observed in this case had not been previously reported in relation to a CEPA.

There was a further deterioration in the patient’s symptoms, although she did not experience any nausea, vomiting or headaches. On examination, her Glasgow coma score was 15 and, apart from the pre-existing left facial nerve palsy, she had no motor deficit. The patient was admitted under the neurosurgeons and underwent an endoscopic third ventriculostomy and biopsy of the pineal region lesion with a free-hand technique and an Aesculap endoscopy kit (MINOP® InVent, Aesculap, B. Braun Medical Ltd., Sheffield, UK) via a right frontal pre-coronal burr hole. A pale pink rounded mass was seen suspended in the posterior part of the third ventricle, overhanging the cerebral aqueduct. Tumour and cerebrospinal fluid samples were sent intraoperatively. There were no perioperative complications and the patient was discharged home on the third postoperative day.

**Figure 4.**
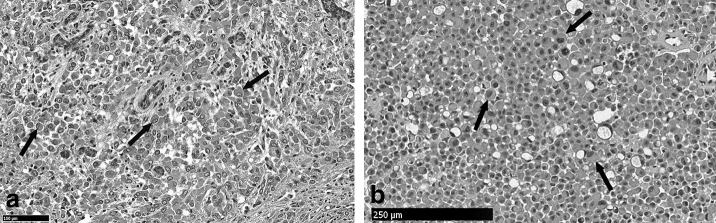
(a) H and E-labelled section (20× magnification) of the primary parotid tumour demonstrating cells with eccentrically placed nuclei consistent with plasmacytoid morphology (black arrows). (b) Magnified H and E-labelled section (10× magnification) from the pineal tumour biopsy demonstrating cells with a plasmacytoid morphology (black arrows) that are analogous to those demonstrated within the parotid gland tumour. H and E, haematoxylin and eosin.

Histological analysis of the pineal tumour determined conclusively that the lesion was not a primary brain malignancy or germ cell tumour, but a metastatic carcinoma. The immunoprofile combination of CK7-positive and CK20-negative was suggestive of salivary gland tumour metastasis. The gross cystic disease fluid protein-15 marker was also identified, and although it is usually expressed in breast carcinomas, its appearance has also been reported in salivary duct carcinomas.^1^ In this case, when the histology from the pineal gland was compared with the original histology, the intracranial lesion was consistent with a metastatic adenocarcinoma from the parotid gland (Figure 4a,b).

**Figure 5.**
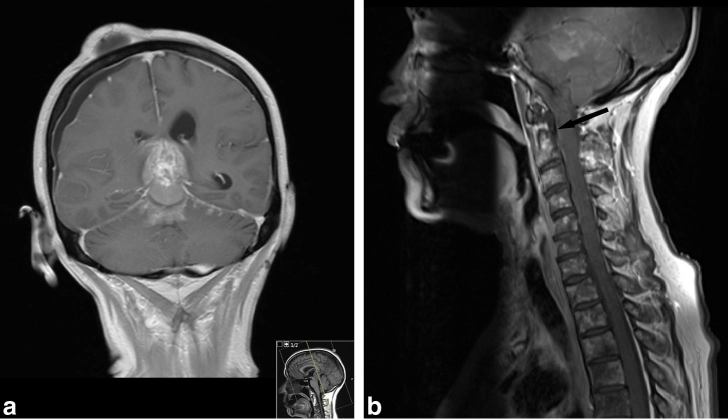
(a) Coronal gadolinium-enhanced *T*_1_ weighted MRI demonstrating progression of the leptomeningeal disease along the superior cerebellar folia. The pineal region tumour is also demonstrated but the hydrocephalus has resolved after ventriculostomy. The shallow right subdural and subgaleal collections are post-procedural. (b) Sagittal gadolinium-enhanced *T*_1_ weighted MRI demonstrating the posterior fossa leptomeningeal enhancement as well as the meningeal enhancement along the anterior cervical spinal cord (black arrow). Also the widespread abnormal bone marrow signal within the vertebral bodies in keeping with metastatic deposits is noteworthy.

Although the patient was neurologically intact at her next follow-up appointment, and the ventricular system had been successfully decompressed, the contrast-enhanced MRI demonstrated that the leptomeningeal enhancement had progressed dramatically and now extended throughout the neuraxis (Figure 5a,b). There was further rapid deterioration in the patient’s condition and she subsequently died.

## Discussion

Pleomorphic adenoma (PA) is the most common non-malignant tumour to affect the salivary glands, usually involving the parotid glands and less commonly the submandibular and other minor salivary glands, as well as the lacrimal glands.^2^ Although benign, it has been reported that as many as 25% of primary and recurrent PAs undergo carcinomatous transformation into CEPA if left untreated.^2,3^ The carcinomatous component is usually adenocarcinoma, not otherwise specified, or salivary duct carcinoma.^1^ The other rarer malignant subsets associated with PAs are carcinosarcoma, which comprises both the epithelial and mesenchymal components of PA, and metastasizing PA (metastatic foci of benign PA).^2,4^ Malignant degeneration should be suspected in lesions that appear infiltrative with an irregular contour on imaging. Ultrasound evaluation of the cervical lymph nodes is also vital in identifying any evidence of local lymphatic dissemination.

CEPAs comprise approximately 12% of all malignant salivary gland neoplasms, usually occurring in the sixth to eighth decades of life.^2–4^ Although often asymptomatic, they most commonly present as a firm mass in the parotid region. The patients may also present with facial nerve palsy owing to deep parotid lobe invasion involving the facial nerve, a finding that should raise the suspicion for malignant degeneration in patients with PA. Transformation into CEPA has been linked to an increased pre-operative duration of a PA.^3^

Local and regional surgical resection followed by radiotherapy is the most recognized appropriate treatment in cases of high-grade CEPA, although there are no standardized treatment protocols.^2,3,5,6^ MRI is the imaging modality of choice for post-treatment follow-up of salivary gland neoplasms, with PET/CT being invaluable for the assessment of suspected recurrence and disseminated disease.^7^

CEPAs are aggressive malignancies with high rates of recurrence and metastases. Owing to the limited number of cases and the variability in histological and radiological staging, survival rates in these patients vary but are regularly reported as less than 1 year.^3,4^ Zhao and colleagues^5^ demonstrated an overall 5-year survival of approximately 50% in a study of 51 patients with CEPA, who were treated with curative intent.

Regional and distant metastases from CEPA appear to occur with an equivalent frequency.^6^ The histological grade of the primary tumour, local invasiveness and cervical lymph node involvement are all important prognostic factors, the latter is recognized as the most reliable feature in predicting recurrence or metastasis.^5^ In this case, although there was no extracapsular extension of the lesion, the tumour was high-grade with multiple cervical lymph nodes also involved. Distant metastases are usually to the lungs and bones, with renal, hepatic and splenic metastases also rarely reported.^4–6^ Intracranial or spinal metastatic spread is exceptionally rare, with only very few cases reported in the literature. Sheedy et al^2^ reported a case of biopsy-proven metastases from CEPA of a parotid primary tumour affecting the brain and intramedullary spinal cord, which they believed to be the first reported case of its kind at the time. Ahn and colleagues^8^ have reported intracranial and spinal metastases from a lacrimal gland CEPA, with recurrent primary tumour directly extending into the cavernous sinus with leptomeningeal deposits also identified. Our case is exceptional in that, to our knowledge, a pineal gland metastasis from a distant CEPA has never been reported. The presence of a metastatic deposit within the cerebellopontine angle from this primary lesion has also not been previously described. The intracranial and spinal leptomeningeal disease, which rapidly progressed in our patient, has only previously been documented in the report by Ahn et al,^8^ indicating that this is also a remarkably rare finding.

Overall, the pineal gland is an unusual location for metastatic spread, with many cases discovered incidentally at autopsy: reports have indicated that there is a prevalence of 0.4–3.8% in patients with solid tumours.^9^ There are few cases of symptomatic pineal region metastases reported in the literature; however, a case reported by Nemoto et al^10^ of a patient with an isolated pineal region metastasis from lung adenocarcinoma also presented with obstructive hydrocephalus. As in our case, there was an indolent clinical course, with little in the way of examination findings and no evidence of raised intracranial pressure. The limited literature available documents that the most common primary tumour site for spread to the pineal gland is the lungs, with other primaries including breasts, malignant melanoma and kidneys also cited.^9,10^ Pineal metastasis from a salivary gland neoplasm has not, to the best of our knowledge, been reported in the literature.

Although this is an extremely rare case report of a pineal gland metastasis, with a cerebellopontine angle deposit and leptomeningeal spread, from a parotid CEPA, it serves to demonstrate how aggressively this primary tumour can behave, as well as serve to illustrate the diagnostic dilemmas that the clinical team may face. A strong, collaborative effort on the part of the multi-disciplinary team looking after these patients is key to the diagnosis and effective treatment of these challenging cases.

## Learning points

1. There is an uncommon yet significant risk of PAs undergoing malignant transformation, particularly if untreated. As such, they should be considered as premalignant conditions.
2. CEPA behaves unpredictably but has high rates of recurrence and metastases, and the discovery of involved lymph nodes is strongly regarded as a poor prognostic sign.
3. Utilization of a variety of imaging modalities in the management of CEPA is strongly recommended. Ultrasound ± needle aspiration or biopsy, MRI and PET/CT investigations will aid in the definitive evaluation of recurrence at the surgical bed, confirmation of local lymph node involvement or distant metastatic spread, and the suspected complications of disease progression.
4. Intracranial metastases are extremely rare in salivary gland tumours and there needs to be close collaboration between clinicians, radiologists and pathologists in ensuring timely diagnosis and treatment.
5. Although an unusual entity, metastases should be a differential diagnosis for any patient with a known malignancy who presents with a pineal region tumour.

## Consent

Informed consent could not be given by the patient as she was deceased at the time of writing this case report. This could not be obtained from the next of kin or a family member despite exhaustive attempts to contact them.
